# The complete mitochondrial genome and phylogenetic analysis of the ocellated angelshark: *Squatina tergocellatoides* Chen, 1963

**DOI:** 10.1080/23802359.2022.2134747

**Published:** 2022-10-27

**Authors:** Chunbin Jia, Binbin Shan, Yan Liu, Changping Yang, Liangming Wang, Dianrong Sun

**Affiliations:** a Shenzhen Fisheries Development Research Center, Shenzhen, China; b Key Laboratory of Marine Ranching, Ministry of Agriculture Rural Affairs, Guangzhou, China; c Guangdong Provincial Key Laboratory of Fishery Ecology and Environment, Guangzhou, China; d South China Sea Fisheries Research Institute, Chinese Academy of Fisheries Sciences, Guangzhou, China

**Keywords:** *Squatina tergocellatoides*, angelshark, mitogenome, Squatinidae

## Abstract

The ocellated angelshark (*Squatina tergocellatoides* Chen, 1963) is a threatened shark within the family Squatinidae. In the present study, we reported the mitochondrial genome sequence of the ocellated angelshark. The complete mitochondrial genome is 16,683 bp in length and contains 37 mitochondrial genes and a control region as similar to most fishes. In addition, we constructed a maximum-likelihood phylogenetic tree of *S. tergocellatoides* and its relative species. This work will provide molecular data for further studies on *S. tergocellatoides*.

Among the 16 valid species of the genus *Squatina*, four species were reported in the western North Pacific, including *S. tergocellatoides*, *S. formosa*, *S. japonica*, and *S. nebulosa* (Compagno et al. 2005). Due to bycatch and destruction of habitat, ocellated angelshark *S. tergocellatoides* population have decreased (Figure 1). In 2020, *S. tergocellatoides* has been assessed for The IUCN Red List of Threatened Species as Endangered under criteria A2d (Rigby et al. 2020). However, the genetic resource of *S. tergocellatoides* is still limited. Hence, we sequenced and reported the complete mitogenome sequence of *S. tergocellatoides* here. Our work will provide molecular data for further studies of *S. tergocellatoides*.

**Figure 1.**
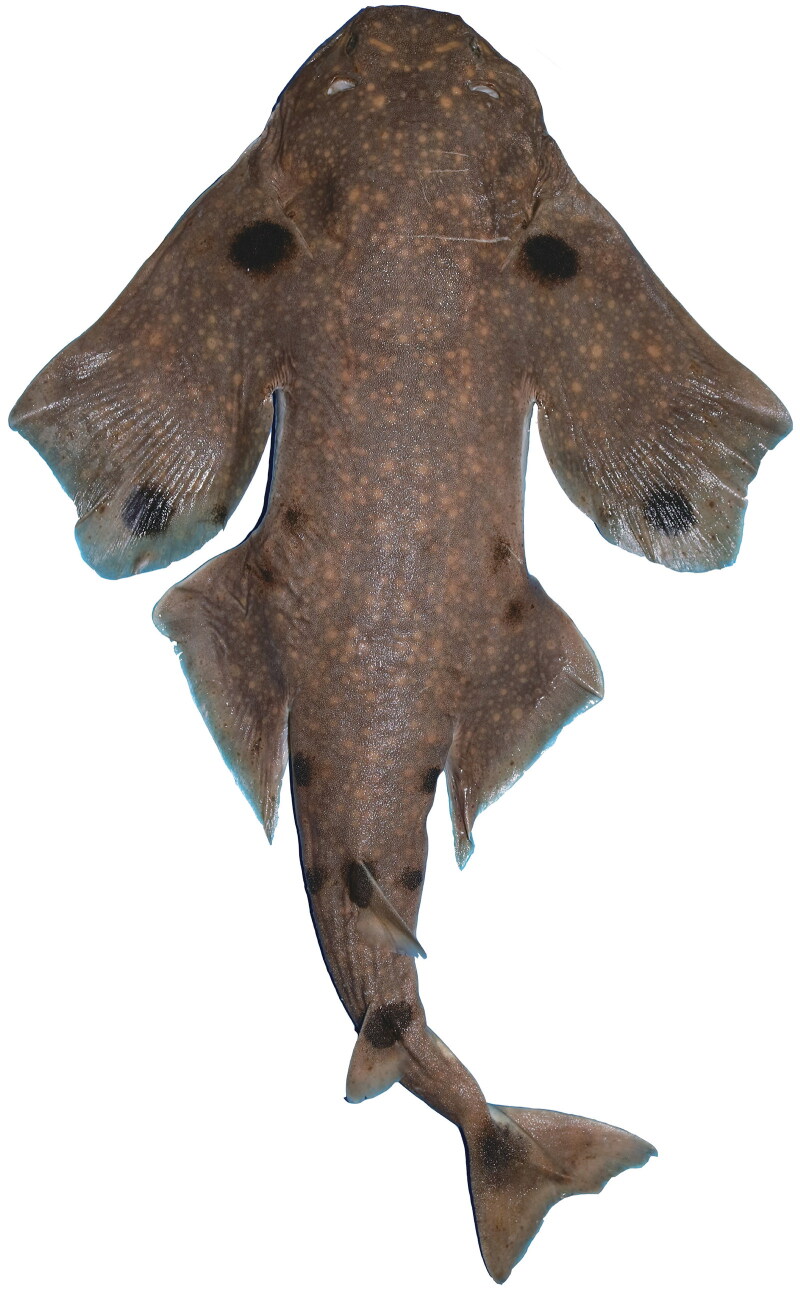
Squatina tergocellatoides.

Tissue samples (muscle) from a *S. tergocellatoides* specimen from the South China Sea (N 118.37°, E 22.83°) were collected in 23 September 2018. Then, the sample was preserved in our laboratory. Genomic DNA of *S. tergocellatoides* was extracted using a Tiangen marine tissue genomic DNA Extraction Kit. The mitogenome of *S. tergocellatoides* was sequenced on an Illumina HiSeq 4000 platform (Table S1). The specimen and its DNA were deposited at Key Laboratory of Marine Ranching, Ministry of Agriculture and Rural Affairs, PR China (BB. Shan, shanbinbin@yeah.net) under the voucher number *Squatina_tergocellatoides*_SCS_01.

After trimming and assembly, we obtained a mitochondrial genome sequence with a total length of 16,683 bp (Figure 2). We annotated the mitogenome by using MITOS2, and identified 22 transfer RNA (tRNA) genes (1499), 13 protein-coding genes (11,435 bp), two ribosomal RNA (rRNA) genes (2603), and a non-coding AT-rich region (1146) (Bernt et al. 2013). The nucleotide composition of the mitogenome is: 13.4% G, 23.1% C, 31.1% A, and 32.4% T, the composition showed an anti-G bias like other fishes (Miya et al. 2001). Furthermore, we constructed a maximum-likelihood phylogenetic tree based on protein-coding genes sequences of the *S. tergocellatoides* and other related sharks (Figure 3). The selected nucleotide sequence model was GTR + R6 + F (Posada and Crandall 1998). The topological structure of the phylogenetic tree shows that *S. tergocellatoides*, *S. nebulosa*, *S. formosa*, and *S. japonica* in genus *Squatina*, have a close relationship. Furthermore, the four species cluster into a sister group to *S. squatina* and *S. aculeata.* The result of our study is an important resource for further genetic studies of *S. tergocellatoides*.

**Figure 2.**
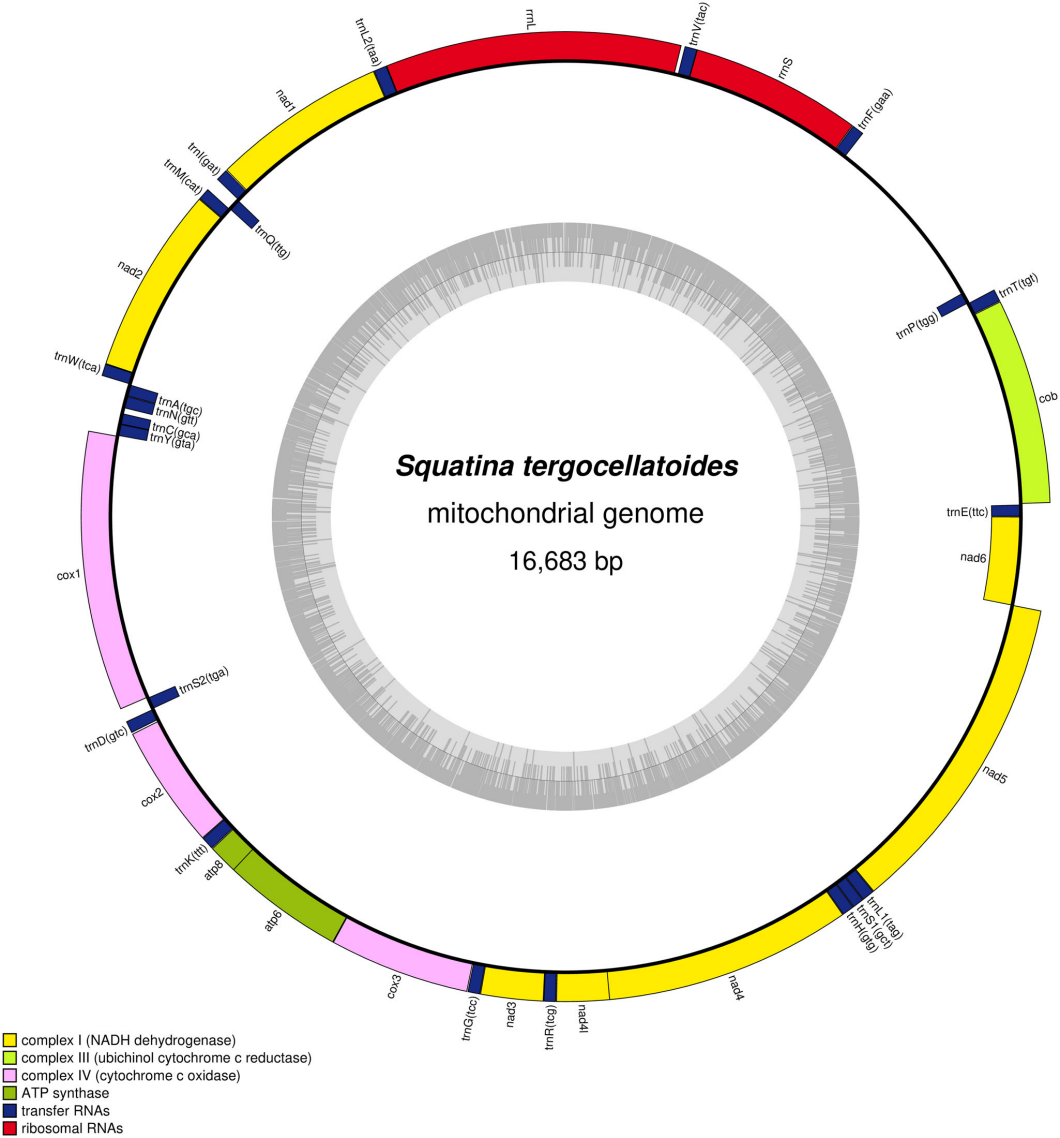
Mitogenome pattern map of Squatina tergocellatoides.

**Figure 3.**
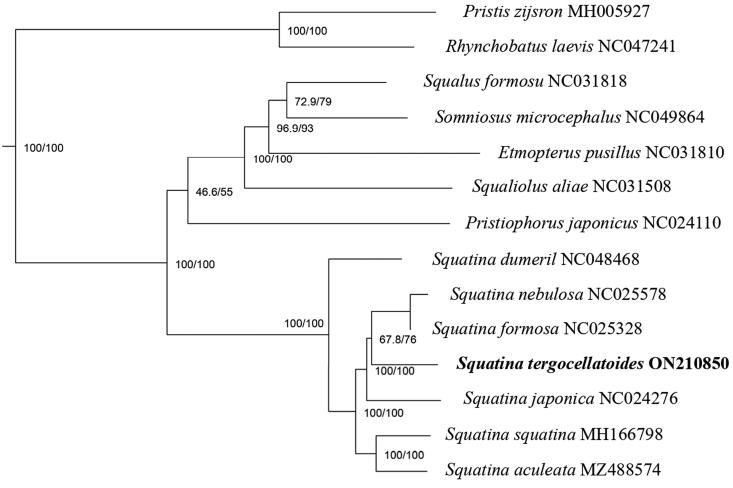
The maximum-likelihood phylogenetic tree of S. tergocellatoides and its relative species.

In the present study, we sequenced and assembled the mitochondrial genome of *S. tergocellatoides*. We annotated the genes and estimated base compositions of the mitochondrial genome. In addition, we constructed a phylogenetic tree using the maximum-likelihood method based on the 13 protein-coding genes of *S. tergocellatoides* and other species. We expect that the results of the present study will facilitate further investigations on the molecular evolution and conservation biology of *S. tergocellatoides.*

## Supplementary Material

Supplemental Material

## Data Availability

The genome sequence data that support the findings of this study are openly available in GenBank of NCBI at https://www.ncbi.nlm.nih.gov/ under accession no. ON210850. The associated BioProject, SRA, and Bio-Sample numbers are PRJNA835906, SRR19134635, and SAMN28125088, respectively.
