# Supplementary material for: The complete mitochondrial genome and phylogenetic analysis of the ocellated angelshark: Squatina tergocellatoides Chen, 1963
Source: Mitochondrial DNA B Resour. 2022 Oct 27;7(10):1851–3. doi: 10.1080/23802359.2022.2134747 (PMC9621291; doi:10.1080/23802359.2022.2134747)
Supplement: Supplemental Material [file TMDN_A_2134747_SM7245.docx]

Table S1 Summary statistics of sequencing data in the present study

| Sample name | *Squatina tergocellatoides* |
| --- | --- |
| Length (bp) | 150 |
| Raw reads | 30,202,576 |
| Raw bases (bp) | 4,530,386,400 |
| Clean reads | 29,853,218 |
| Clean bases (bp) | 4,477,982,700 |
| GC (%) | 51.02 |
| Q20 (%) | 92.56 |
| Q30 (%) | 86.59 |
